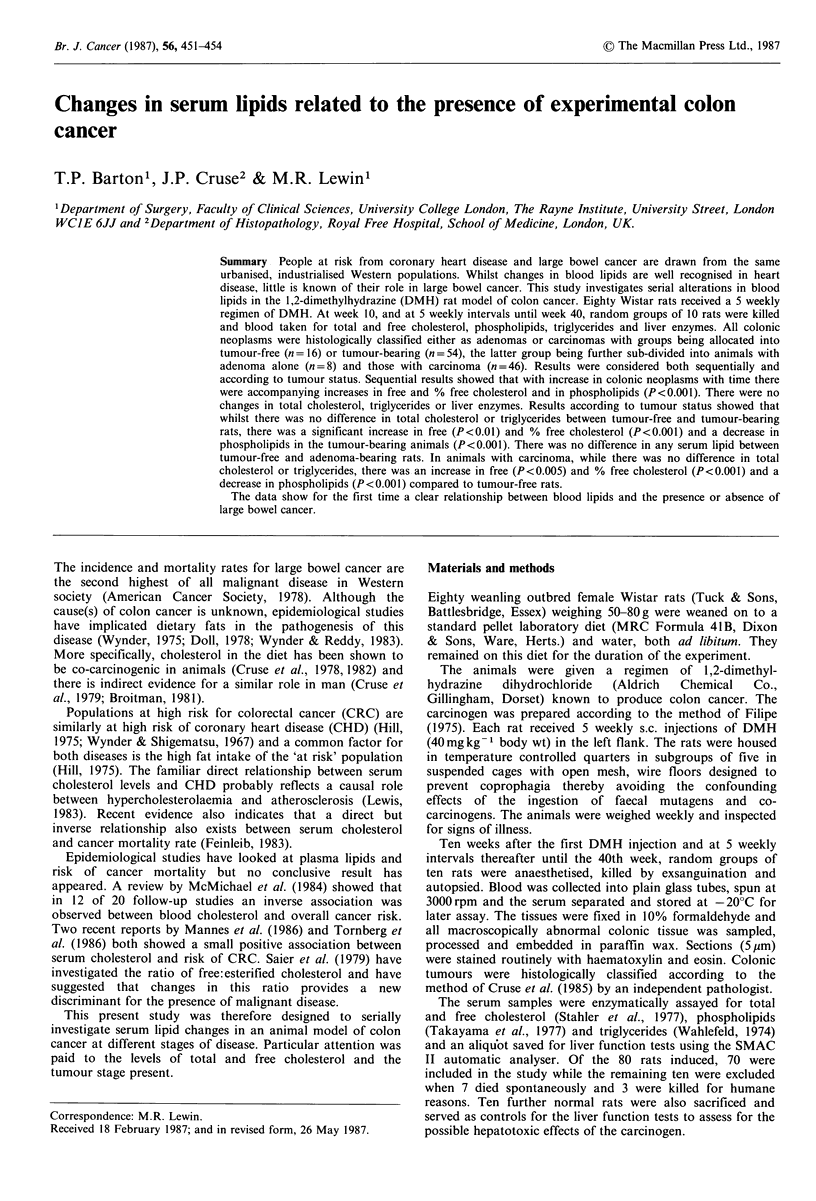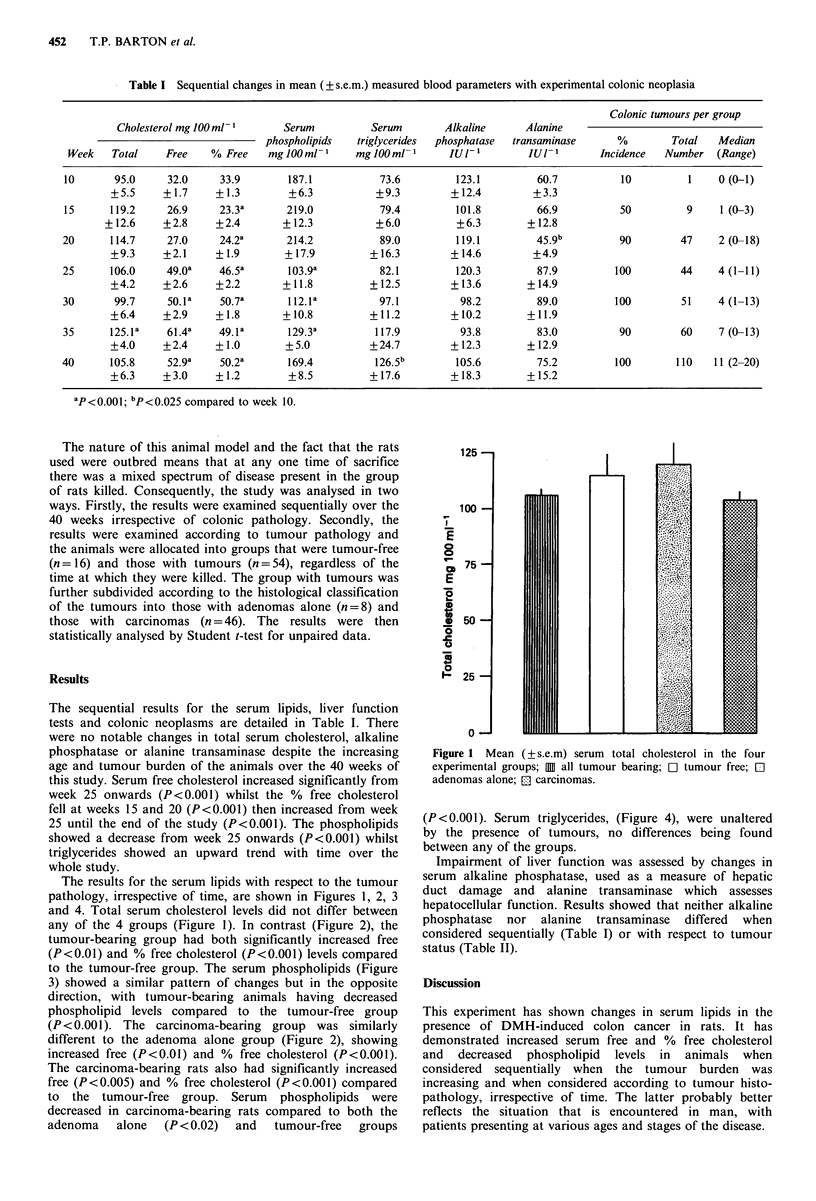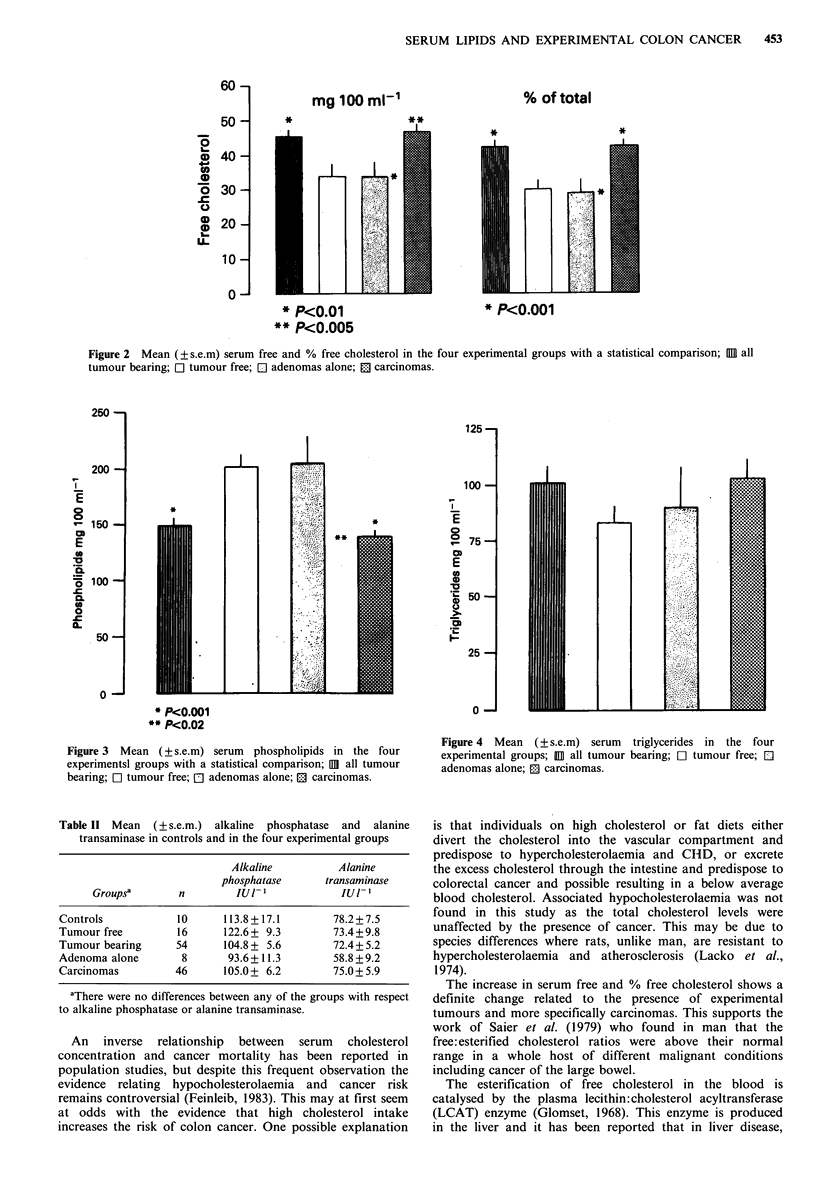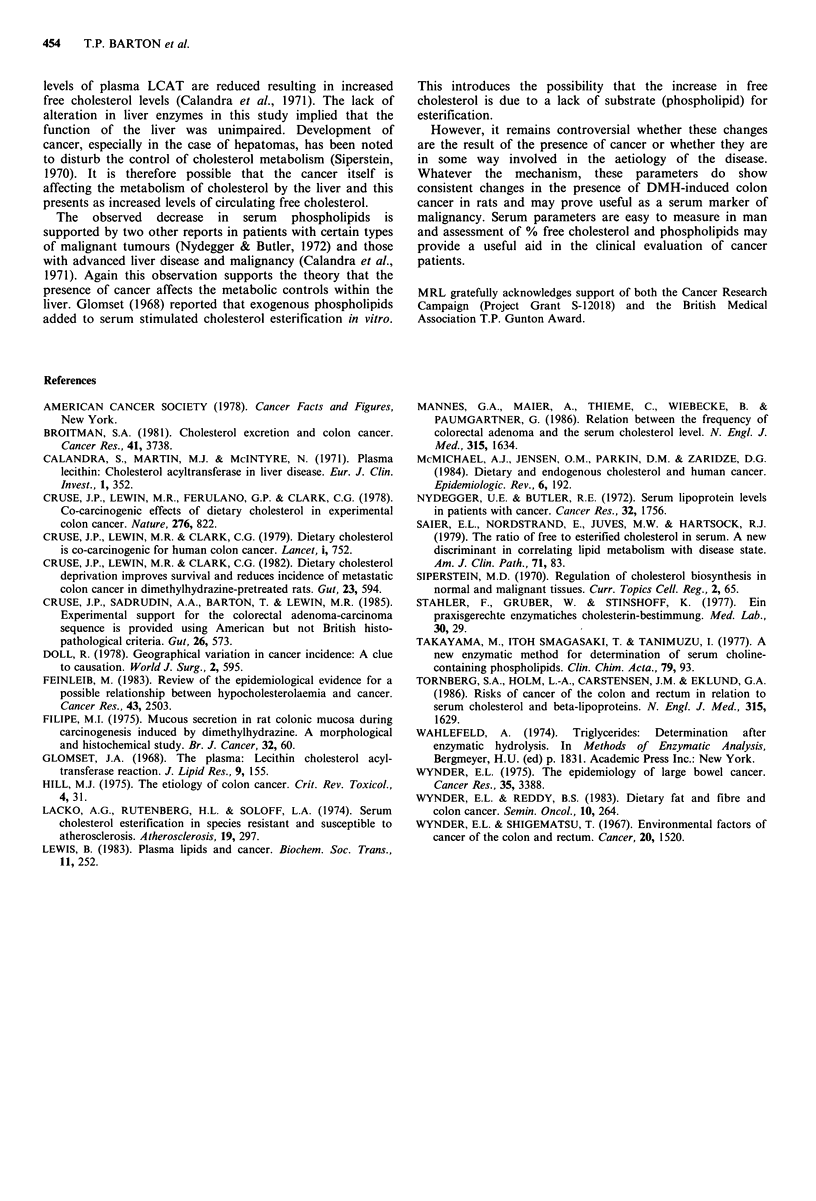# Changes in serum lipids related to the presence of experimental colon cancer.

**DOI:** 10.1038/bjc.1987.222

**Published:** 1987-10

**Authors:** T. P. Barton, J. P. Cruse, M. R. Lewin

**Affiliations:** Department of Surgery, Faculty of Clinical Sciences, University College, London, UK.

## Abstract

People at risk from coronary heart disease and large bowel cancer are drawn from the same urbanised, industrialised Western populations. Whilst changes in blood lipids are well recognised in heart disease, little is known of their role in large bowel cancer. This study investigates serial alterations in blood lipids in the 1,2-dimethylhydrazine (DMH) rat model of colon cancer. Eighty Wistar rats received a 5 weekly regimen of DMH. At week 10, and at 5 weekly intervals until week 40, random groups of 10 rats were killed and blood taken for total and free cholesterol, phospholipids, triglycerides and liver enzymes. All colonic neoplasms were histologically classified either as adenomas or carcinomas with groups being allocated into tumour-free (n = 16) or tumour-bearing (n = 54), the latter group being further sub-divided into animals with adenoma alone (n = 8) and those with carcinoma (n = 46). Results were considered both sequentially and according to tumour status. Sequential results showed that with increase in colonic neoplasms with time there were accompanying increases in free and % free cholesterol and in phospholipids (P less than 0.001). There were no changes in total cholesterol, triglycerides or liver enzymes. Results according to tumour status showed that whilst there was no difference in total cholesterol or triglycerides between tumour-free and tumour-bearing rats, there was a significant increase in free (P less than 0.01) and % free cholesterol (P less than 0.001) and a decrease in phospholipids in the tumour-bearing animals (P less than 0.001). There was no difference in any serum lipid between tumour-free and adenoma-bearing rats. In animals with carcinoma, while there was no difference in total cholesterol or triglycerides, there was an increase in free (P less than 0.005) and % free cholesterol (P less than 0.001) and a decrease in phospholipids (P less than 0.001) compared to tumour-free rats. The data show for the first time a clear relationship between blood lipids and the presence or absence of large bowel cancer.


*Br. J. Cancer* (1987), **56**, 451–454 © The Macmillan Press Ltd., 1987

### Changes in serum lipids related to the presence of experimental colon cancer

T.P. Barton[1], J.P. Cruse[2] & M.R. Lewin[1]

[1]*Department of Surgery, Faculty of Clinical Sciences, University College London, The Rayne Institute, University Street, London WC1E 6JJ and* [2]*Department of Histopathology, Royal Free Hospital, School of Medicine, London, UK.*

**Summary** People at risk from coronary heart disease and large bowel cancer are drawn from the same urbanised, industrialised Western populations. Whilst changes in blood lipids are well recognised in heart disease, little is known of their role in large bowel cancer. This study investigates serial alterations in blood lipids in the 1,2-dimethylhydrazine (DMH) rat model of colon cancer. Eighty Wistar rats received a 5 weekly regimen of DMH. At week 10, and at 5 weekly intervals until week 40, random groups of 10 rats were killed and blood taken for total and free cholesterol, phospholipids, triglycerides and liver enzymes. All colonic neoplasms were histologically classified either as adenomas or carcinomas with groups being allocated into tumour-free ($n=16$) or tumour-bearing ($n=54$), the latter group being further sub-divided into animals with adenoma alone ($n=8$) and those with carcinoma ($n=46$). Results were considered both sequentially and according to tumour status. Sequential results showed that with increase in colonic neoplasms with time there were accompanying increases in free and % free cholesterol and in phospholipids ($P<0.001$). There were no changes in total cholesterol, triglycerides or liver enzymes. Results according to tumour status showed that whilst there was no difference in total cholesterol or triglycerides between tumour-free and tumour-bearing rats, there was a significant increase in free ($P<0.01$) and % free cholesterol ($P<0.001$) and a decrease in phospholipids in the tumour-bearing animals ($P<0.001$). There was no difference in any serum lipid between tumour-free and adenoma-bearing rats. In animals with carcinoma, while there was no difference in total cholesterol or triglycerides, there was an increase in free ($P<0.005$) and % free cholesterol ($P<0.001$) and a decrease in phospholipids ($P<0.001$) compared to tumour-free rats.

The data show for the first time a clear relationship between blood lipids and the presence or absence of large bowel cancer.

The incidence and mortality rates for large bowel cancer are the second highest of all malignant disease in Western society (American Cancer Society, 1978). Although the cause(s) of colon cancer is unknown, epidemiological studies have implicated dietary fats in the pathogenesis of this disease (Wynder, 1975; Doll, 1978; Wynder & Reddy, 1983). More specifically, cholesterol in the diet has been shown to be co-carcinogenic in animals (Cruse *et al.*, 1978, 1982) and there is indirect evidence for a similar role in man (Cruse *et al.*, 1979; Broitman, 1981).

Populations at high risk for colorectal cancer (CRC) are similarly at high risk of coronary heart disease (CHD) (Hill, 1975; Wynder & Shigematsu, 1967) and a common factor for both diseases is the high fat intake of the 'at risk' population (Hill, 1975). The familiar direct relationship between serum cholesterol levels and CHD probably reflects a causal role between hypercholesterolaemia and atherosclerosis (Lewis, 1983). Recent evidence also indicates that a direct but inverse relationship also exists between serum cholesterol and cancer mortality rate (Feinleib, 1983).

Epidemiological studies have looked at plasma lipids and risk of cancer mortality but no conclusive result has appeared. A review by McMichael *et al.* (1984) showed that in 12 of 20 follow-up studies an inverse association was observed between blood cholesterol and overall cancer risk. Two recent reports by Mannes *et al.* (1986) and Tornberg *et al.* (1986) both showed a small positive association between serum cholesterol and risk of CRC. Saier *et al.* (1979) have investigated the ratio of free:esterified cholesterol and have suggested that changes in this ratio provides a new discriminant for the presence of malignant disease.

This present study was therefore designed to serially investigate serum lipid changes in an animal model of colon cancer at different stages of disease. Particular attention was paid to the levels of total and free cholesterol and the tumour stage present.

Correspondence: M.R. Lewin.
Received 18 February 1987; and in revised form, 26 May 1987.

### Materials and methods

Eighty weanling outbred female Wistar rats (Tuck & Sons, Battlesbridge, Essex) weighing 50–80 g were weaned on to a standard pellet laboratory diet (MRC Formula 41B, Dixon & Sons, Ware, Herts.) and water, both *ad libitum*. They remained on this diet for the duration of the experiment.

The animals were given a regimen of 1,2-dimethylhydrazine dihydrochloride (Aldrich Chemical Co., Gillingham, Dorset) known to produce colon cancer. The carcinogen was prepared according to the method of Filipe (1975). Each rat received 5 weekly s.c. injections of DMH ($40\,mg\,kg^{-1}$ body wt) in the left flank. The rats were housed in temperature controlled quarters in subgroups of five in suspended cages with open mesh, wire floors designed to prevent coprophagia thereby avoiding the confounding effects of the ingestion of faecal mutagens and co-carcinogens. The animals were weighed weekly and inspected for signs of illness.

Ten weeks after the first DMH injection and at 5 weekly intervals thereafter until the 40th week, random groups of ten rats were anaesthetised, killed by exsanguination and autopsied. Blood was collected into plain glass tubes, spun at 3000 rpm and the serum separated and stored at $-20°C$ for later assay. The tissues were fixed in 10% formaldehyde and all macroscopically abnormal colonic tissue was sampled, processed and embedded in paraffin wax. Sections ($5\,\mu m$) were stained routinely with haematoxylin and eosin. Colonic tumours were histologically classified according to the method of Cruse *et al.* (1985) by an independent pathologist.

The serum samples were enzymatically assayed for total and free cholesterol (Stahler *et al.*, 1977), phospholipids (Takayama *et al.*, 1977) and triglycerides (Wahlefeld, 1974) and an aliquot saved for liver function tests using the SMAC II automatic analyser. Of the 80 rats induced, 70 were included in the study while the remaining ten were excluded when 7 died spontaneously and 3 were killed for humane reasons. Ten further normal rats were also sacrificed and served as controls for the liver function tests to assess for the possible hepatotoxic effects of the carcinogen.

**Table I** Sequential changes in mean (±s.e.m.) measured blood parameters with experimental colonic neoplasia

| Week | Cholesterol mg 100 ml⁻¹ Total | Free | % Free | Serum phospholipids mg 100 ml⁻¹ | Serum triglycerides mg 100 ml⁻¹ | Alkaline phosphatase IU l⁻¹ | Alanine transaminase IU l⁻¹ | Colonic tumours per group % Incidence | Total Number | Median (Range) |
|---|---|---|---|---|---|---|---|---|---|---|
| 10 | 95.0 ±5.5 | 32.0 ±1.7 | 33.9 ±1.3 | 187.1 ±6.3 | 73.6 ±9.3 | 123.1 ±12.4 | 60.7 ±3.3 | 10 | 1 | 0 (0–1) |
| 15 | 119.2 ±12.6 | 26.9 ±2.8 | 23.3[a] ±2.4 | 219.0 ±12.3 | 79.4 ±6.0 | 101.8 ±6.3 | 66.9 ±12.8 | 50 | 9 | 1 (0–3) |
| 20 | 114.7 ±9.3 | 27.0 ±2.1 | 24.2[a] ±1.9 | 214.2 ±17.9 | 89.0 ±16.3 | 119.1 ±14.6 | 45.9[b] ±4.9 | 90 | 47 | 2 (0–18) |
| 25 | 106.0 ±4.2 | 49.0[a] ±2.6 | 46.5[a] ±2.2 | 103.9[a] ±11.8 | 82.1 ±12.5 | 120.3 ±13.6 | 87.9 ±14.9 | 100 | 44 | 4 (1–11) |
| 30 | 99.7 ±6.4 | 50.1[a] ±2.9 | 50.7[a] ±1.8 | 112.1[a] ±10.8 | 97.1 ±11.2 | 98.2 ±10.2 | 89.0 ±11.9 | 100 | 51 | 4 (1–13) |
| 35 | 125.1[a] ±4.0 | 61.4[a] ±2.4 | 49.1[a] ±1.0 | 129.3[a] ±5.0 | 117.9 ±24.7 | 93.8 ±12.3 | 83.0 ±12.9 | 90 | 60 | 7 (0–13) |
| 40 | 105.8 ±6.3 | 52.9[a] ±3.0 | 50.2[a] ±1.2 | 169.4 ±8.5 | 126.5[b] ±17.6 | 105.6 ±18.3 | 75.2 ±15.2 | 100 | 110 | 11 (2–20) |

[a]$P<0.001$; [b]$P<0.025$ compared to week 10.

The nature of this animal model and the fact that the rats used were outbred means that at any one time of sacrifice there was a mixed spectrum of disease present in the group of rats killed. Consequently, the study was analysed in two ways. Firstly, the results were examined sequentially over the 40 weeks irrespective of colonic pathology. Secondly, the results were examined according to tumour pathology and the animals were allocated into groups that were tumour-free ($n=16$) and those with tumours ($n=54$), regardless of the time at which they were killed. The group with tumours was further subdivided according to the histological classification of the tumours into those with adenomas alone ($n=8$) and those with carcinomas ($n=46$). The results were then statistically analysed by Student *t*-test for unpaired data.

### Results

The sequential results for the serum lipids, liver function tests and colonic neoplasms are detailed in Table I. There were no notable changes in total serum cholesterol, alkaline phosphatase or alanine transaminase despite the increasing age and tumour burden of the animals over the 40 weeks of this study. Serum free cholesterol increased significantly from week 25 onwards ($P<0.001$) whilst the % free cholesterol fell at weeks 15 and 20 ($P<0.001$) then increased from week 25 until the end of the study ($P<0.001$). The phospholipids showed a decrease from week 25 onwards ($P<0.001$) whilst triglycerides showed an upward trend with time over the whole study.

The results for the serum lipids with respect to the tumour pathology, irrespective of time, are shown in Figures 1, 2, 3 and 4. Total serum cholesterol levels did not differ between any of the 4 groups (Figure 1). In contrast (Figure 2), the tumour-bearing group had both significantly increased free ($P<0.01$) and % free cholesterol ($P<0.001$) levels compared to the tumour-free group. The serum phospholipids (Figure 3) showed a similar pattern of changes but in the opposite direction, with tumour-bearing animals having decreased phospholipid levels compared to the tumour-free group ($P<0.001$). The carcinoma-bearing group was similarly different to the adenoma alone group (Figure 2), showing increased free ($P<0.01$) and % free cholesterol ($P<0.001$). The carcinoma-bearing rats also had significantly increased free ($P<0.005$) and % free cholesterol ($P<0.001$) compared to the tumour-free group. Serum phospholipids were decreased in carcinoma-bearing rats compared to both the adenoma alone ($P<0.02$) and tumour-free groups

**Figure 1** Mean (±s.e.m) serum total cholesterol in the four experimental groups; ▥ all tumour bearing; □ tumour free; ⬚ adenomas alone; ▩ carcinomas.

($P<0.001$). Serum triglycerides, (Figure 4), were unaltered by the presence of tumours, no differences being found between any of the groups.

Impairment of liver function was assessed by changes in serum alkaline phosphatase, used as a measure of hepatic duct damage and alanine transaminase which assesses hepatocellular function. Results showed that neither alkaline phosphatase nor alanine transaminase differed when considered sequentially (Table I) or with respect to tumour status (Table II).

### Discussion

This experiment has shown changes in serum lipids in the presence of DMH-induced colon cancer in rats. It has demonstrated increased serum free and % free cholesterol and decreased phospholipid levels in animals when considered sequentially when the tumour burden was increasing and when considered according to tumour histopathology, irrespective of time. The latter probably better reflects the situation that is encountered in man, with patients presenting at various ages and stages of the disease.

**Figure 2** Mean (±s.e.m) serum free and % free cholesterol in the four experimental groups with a statistical comparison; ▥ all tumour bearing; □ tumour free; ▨ adenomas alone; ▩ carcinomas.

**Figure 3** Mean (±s.e.m) serum phospholipids in the four experimentsl groups with a statistical comparison; ▥ all tumour bearing; □ tumour free; ▨ adenomas alone; ▩ carcinomas.

**Figure 4** Mean (±s.e.m) serum triglycerides in the four experimental groups; ▥ all tumour bearing; □ tumour free; ▨ adenomas alone; ▩ carcinomas.

**Table II** Mean (±s.e.m.) alkaline phosphatase and alanine transaminase in controls and in the four experimental groups

| *Groups*[a] | *n* | *Alkaline phosphatase IU l$^{-1}$* | *Alanine transaminase IU l$^{-1}$* |
|---|---|---|---|
| Controls | 10 | 113.8±17.1 | 78.2±7.5 |
| Tumour free | 16 | 122.6± 9.3 | 73.4±9.8 |
| Tumour bearing | 54 | 104.8± 5.6 | 72.4±5.2 |
| Adenoma alone | 8 | 93.6±11.3 | 58.8±9.2 |
| Carcinomas | 46 | 105.0± 6.2 | 75.0±5.9 |

[a]There were no differences between any of the groups with respect to alkaline phosphatase or alanine transaminase.

An inverse relationship between serum cholesterol concentration and cancer mortality has been reported in population studies, but despite this frequent observation the evidence relating hypocholesterolaemia and cancer risk remains controversial (Feinleib, 1983). This may at first seem at odds with the evidence that high cholesterol intake increases the risk of colon cancer. One possible explanation is that individuals on high cholesterol or fat diets either divert the cholesterol into the vascular compartment and predispose to hypercholesterolaemia and CHD, or excrete the excess cholesterol through the intestine and predispose to colorectal cancer and possible resulting in a below average blood cholesterol. Associated hypocholesterolaemia was not found in this study as the total cholesterol levels were unaffected by the presence of cancer. This may be due to species differences where rats, unlike man, are resistant to hypercholesterolaemia and atherosclerosis (Lacko *et al.*, 1974).

The increase in serum free and % free cholesterol shows a definite change related to the presence of experimental tumours and more specifically carcinomas. This supports the work of Saier *et al.* (1979) who found in man that the free:esterified cholesterol ratios were above their normal range in a whole host of different malignant conditions including cancer of the large bowel.

The esterification of free cholesterol in the blood is catalysed by the plasma lecithin:cholesterol acyltransferase (LCAT) enzyme (Glomset, 1968). This enzyme is produced in the liver and it has been reported that in liver disease, levels of plasma LCAT are reduced resulting in increased free cholesterol levels (Calandra *et al.*, 1971). The lack of alteration in liver enzymes in this study implied that the function of the liver was unimpaired. Development of cancer, especially in the case of hepatomas, has been noted to disturb the control of cholesterol metabolism (Siperstein, 1970). It is therefore possible that the cancer itself is affecting the metabolism of cholesterol by the liver and this presents as increased levels of circulating free cholesterol.

The observed decrease in serum phospholipids is supported by two other reports in patients with certain types of malignant tumours (Nydegger & Butler, 1972) and those with advanced liver disease and malignancy (Calandra *et al.*, 1971). Again this observation supports the theory that the presence of cancer affects the metabolic controls within the liver. Glomset (1968) reported that exogenous phospholipids added to serum stimulated cholesterol esterification *in vitro*. This introduces the possibility that the increase in free cholesterol is due to a lack of substrate (phospholipid) for esterification.

However, it remains controversial whether these changes are the result of the presence of cancer or whether they are in some way involved in the aetiology of the disease. Whatever the mechanism, these parameters do show consistent changes in the presence of DMH-induced colon cancer in rats and may prove useful as a serum marker of malignancy. Serum parameters are easy to measure in man and assessment of % free cholesterol and phospholipids may provide a useful aid in the clinical evaluation of cancer patients.

MRL gratefully acknowledges support of both the Cancer Research Campaign (Project Grant S-12018) and the British Medical Association T.P. Gunton Award.

### References

AMERICAN CANCER SOCIETY (1978). *Cancer Facts and Figures*, New York.

BROITMAN, S.A. (1981). Cholesterol excretion and colon cancer. *Cancer Res.*, **41**, 3738.

CALANDRA, S., MARTIN, M.J. & McINTYRE, N. (1971). Plasma lecithin: Cholesterol acyltransferase in liver disease. *Eur. J. Clin. Invest.*, **1**, 352.

CRUSE, J.P., LEWIN, M.R., FERULANO, G.P. & CLARK, C.G. (1978). Co-carcinogenic effects of dietary cholesterol in experimental colon cancer. *Nature*, **276**, 822.

CRUSE, J.P., LEWIN, M.R. & CLARK, C.G. (1979). Dietary cholesterol is co-carcinogenic for human colon cancer. *Lancet*, **i**, 752.

CRUSE, J.P., LEWIN, M.R. & CLARK, C.G. (1982). Dietary cholesterol deprivation improves survival and reduces incidence of metastatic colon cancer in dimethylhydrazine-pretreated rats. *Gut*, **23**, 594.

CRUSE, J.P., SADRUDIN, A.A., BARTON, T. & LEWIN, M.R. (1985). Experimental support for the colorectal adenoma-carcinoma sequence is provided using American but not British histopathological criteria. *Gut*, **26**, 573.

DOLL, R. (1978). Geographical variation in cancer incidence: A clue to causation. *World J. Surg.*, **2**, 595.

FEINLEIB, M. (1983). Review of the epidemiological evidence for a possible relationship between hypocholesterolaemia and cancer. *Cancer Res.*, **43**, 2503.

FILIPE, M.I. (1975). Mucous secretion in rat colonic mucosa during carcinogenesis induced by dimethylhydrazine. A morphological and histochemical study. *Br. J. Cancer*, **32**, 60.

GLOMSET, J.A. (1968). The plasma: Lecithin cholesterol acyltransferase reaction. *J. Lipid Res.*, **9**, 155.

HILL, M.J. (1975). The etiology of colon cancer. *Crit. Rev. Toxicol.*, **4**, 31.

LACKO, A.G., RUTENBERG, H.L. & SOLOFF, L.A. (1974). Serum cholesterol esterification in species resistant and susceptible to atherosclerosis. *Atherosclerosis*, **19**, 297.

LEWIS, B. (1983). Plasma lipids and cancer. *Biochem. Soc. Trans.*, **11**, 252.

MANNES, G.A., MAIER, A., THIEME, C., WIEBECKE, B. & PAUMGARTNER, G. (1986). Relation between the frequency of colorectal adenoma and the serum cholesterol level. *N. Engl. J. Med.*, **315**, 1634.

McMICHAEL, A.J., JENSEN, O.M., PARKIN, D.M. & ZARIDZE, D.G. (1984). Dietary and endogenous cholesterol and human cancer. *Epidemiologic. Rev.*, **6**, 192.

NYDEGGER, U.E. & BUTLER, R.E. (1972). Serum lipoprotein levels in patients with cancer. *Cancer Res.*, **32**, 1756.

SAIER, E.L., NORDSTRAND, E., JUVES, M.W. & HARTSOCK, R.J. (1979). The ratio of free to esterified cholesterol in serum. A new discriminant in correlating lipid metabolism with disease state. *Am. J. Clin. Path.*, **71**, 83.

SIPERSTEIN, M.D. (1970). Regulation of cholesterol biosynthesis in normal and malignant tissues. *Curr. Topics Cell. Reg.*, **2**, 65.

STAHLER, F., GRUBER, W. & STINSHOFF, K. (1977). Ein praxisgerechte enzymatiches cholesterin-bestimmung. *Med. Lab.*, **30**, 29.

TAKAYAMA, M., ITOH SMAGASAKI, T. & TANIMUZU, I. (1977). A new enzymatic method for determination of serum choline-containing phospholipids. *Clin. Chim. Acta.*, **79**, 93.

TORNBERG, S.A., HOLM, L.-A., CARSTENSEN, J.M. & EKLUND, G.A. (1986). Risks of cancer of the colon and rectum in relation to serum cholesterol and beta-lipoproteins. *N. Engl. J. Med.*, **315**, 1629.

WAHLEFELD, A. (1974). Triglycerides: Determination after enzymatic hydrolysis. In *Methods of Enzymatic Analysis*, Bergmeyer, H.U. (ed) p. 1831. Academic Press Inc.: New York.

WYNDER, E.L. (1975). The epidemiology of large bowel cancer. *Cancer Res.*, **35**, 3388.

WYNDER, E.L. & REDDY, B.S. (1983). Dietary fat and fibre and colon cancer. *Semin. Oncol.*, **10**, 264.

WYNDER, E.L. & SHIGEMATSU, T. (1967). Environmental factors of cancer of the colon and rectum. *Cancer*, **20**, 1520.